# Response to ‘Time‐course of acid–base regulation at high altitude: A century of insight’ by Andrew R. Steele, Jordan D. Bird and Michael M. Tymko

**DOI:** 10.1113/EP093401

**Published:** 2025-11-20

**Authors:** Axel Kleinsasser, Martin Burtscher, Benedikt Treml, Sasa Rajsic

**Affiliations:** ^1^ Department of Anaesthesiology and Critical Care Medicine Medical University of Innsbruck Innsbruck Austria; ^2^ Department of Sports Sciences University of Innsbruck Innsbruck Austria

## 1 INTRODUCTION

We are very pleased to receive a second letter in response to our recent publication in *Experimental Physiology* (Skalla et al., 2026) and we sincerely appreciate the interest of Steele and coworkers (Steele et al., 2026) in our understanding of acid–base regulation during *early* acclimatisation. Steele and co‐authors present a concise yet short review, accompanied by a detailed figure summarising arterial blood gas findings from the available literature, and raise an important question regarding the genesis of plasma volume contraction at altitude (Steele et al., 2026). Volume contraction in early acclimatisation in our subjects, according to Steele and coworkers’ interpretation, was based on fluid redistribution, and diuresis was primarily due to increased fluid and/or sodium intake (Steele et al., 2026). They further compare our findings with those of Roche and coworkers, who performed a highly controlled hypobaric chamber experiment in nearby Bolzano (Roche et al., 2022) under similar barometric pressure and relative humidity conditions as in our field study (Skalla et al., 2026). We wish to respond to Steele and coworkers’ viewpoints and also address their questioning of the novelty of our findings.

## 2 GENESIS OF PLASMA VOLUME CONTRACTION: DIURESIS OR FLUID REDISTRIBUTION OR A COMBINATION OF BOTH?

Exposure to high altitude can coincide with exposure to low humidity. During our experimental period at Hoher Sonnblick, the relative humidity was around 30%, which is low and likely encouraged increased fluid intake, as was indeed observed. On the first day, our participants drank 1645 mL and had a net balance of −814 mL after day 1. Elevated diuresis and loss of body mass (436 g over 2 days), as well as an increase in haematocrit and haemoglobin on day 2, suggests that diuresis is among the factors that help to restore arterial oxygen content during early acclimatisation. Looking at the data of the Roche experiment, we also see a lag in plasma volume contraction, which became visible after 12 h in hypobaric hypoxia. Moreover, in this elegant experiment, the authors attribute an initial high urinary flow to the osmotic effect of bicarbonaturia (Steele et al., 2026). The same was the case in our experiment. Accordingly, we suggested, ‘during early acclimatization diuresis outweighs fluid shifts in the generation of … plasma volume contraction’ (Skalla et al., 2026).

At no point did we suggest that diuresis was a direct effect of hypoxia, as Steele and coworkers state in their letter (Steele et al., 2026).

However, indirect effects are known, and particularly the initial increase in cardiac output (before volume contraction decreases stroke volume) increases renal blood flow. This seems a very plausible explanation for the diuresis right upon arrival at altitude (Vogel & Harris, 1967). We focused on early acclimatisation during which plasma volume contraction occurs immediately upon arrival at altitude. However, plasma volume contraction becomes more pronounced before plateauing in the first 1–2 weeks (Siebenmann et al., 2017). Taking all of these points into account, diuresis seems a factor in plasma volume contraction during early acclimatisation. Further plasma volume regulation is based on a reduction of total circulating plasma protein (Steele et al., 2026), and this may take some time.

The total fluid intake in our study was approximately 5 L, which is not a large amount for 44 h at 3100 m. Doubts remain whether these 5 L at altitude may explain the increased urinary output as suggested by Steele and coworkers (Steele et al., 2026).

## 3 NOVELTY OF OUR FINDINGS

Steele and coworkers question the novelty of our findings (Steele et al., 2026). We believe that at the time of submission there was no field study with parallel analyses of blood gases, acid–base variables and renal reactivity during staging at 3100 m. This might be a physiological background during acclimatisation days in Namche, Nepal or Lenin Peak basecamp, Kyrgyzstan or other places at a similar altitude and barometric pressure.

Finally, the authors also wish to thank Steele and coworkers for their kind suggestions of future research questions (Steele et al., 2026).

## AUTHOR CONTRIBUTIONS

All authors have read and approved the final version of this manuscript and agree to be accountable for all aspects of the work in ensuring that questions related to the accuracy or integrity of any part of the work are appropriately investigated and resolved. All persons designated as authors qualify for authorship, and all those who qualify for authorship are listed.

## CONFLICT OF INTEREST

The authors declare they have no conflicts of interest.

## FUNDING INFORMATION

No funding was received for this work.
